# Mesenchymal stem cell transplantation alleviated atherosclerosis in systemic lupus erythematosus through reducing MDSCs

**DOI:** 10.1186/s13287-022-03002-y

**Published:** 2022-07-18

**Authors:** Genhong Yao, Jingjing Qi, Xiaojing Li, Xiaojun Tang, Wenchao Li, Weiwei Chen, Nan Xia, Shiying Wang, Lingyun Sun

**Affiliations:** 1 Department of Rheumatology and Immunology, The Affiliated Drum Tower Hospital of Nanjing University Medical School, Nanjing, 210008 China; 2 Department of Immunology, College of Basic Medical Science, Dalian Medical University, Dalian, China

**Keywords:** Systemic lupus erythematosus, Atherosclerosis, Mesenchymal stem cells, Myeloid-derived suppressor cells, Prostaglandin E 2

## Abstract

**Objective:**

The mechanism by which mesenchymal stem cell (MSC) transplantation alleviates atherosclerosis in systemic lupus erythematosus (SLE) remains elusive. In this study, we aim to explore the efficacy and mechanism of MSC in ameliorating atherosclerosis in SLE.

**Methods:**

ApoE^−/−^ and Fas^−/−^ mice on the B6 background were cross-bred to generate SLE mice with atherosclerosis. Myeloid-derived suppressor cells (MDSCs) were sorted and quantified. The apoE^−/−^Fas^−/−^ mice were either treated with anti-Gr antibody or injected with MDSCs. The lupus-like autoimmunity and atherosclerotic lesions were evaluated. Furthermore, the apoE^−/−^Fas^−/−^ mice were transplanted with MSCs and lupus-like autoimmunity and atherosclerotic lesions were assessed.

**Results:**

MDSCs in peripheral blood, spleen, draining lymph nodes increased in apoE^−/−^Fas^−/−^ mice compared with B6 mice. Moreover, the adoptive transfer of MDSCs aggravated both atherosclerosis and SLE pathologies, whereas depleting MDSCs ameliorated those pathologies in apoE^−/−^Fas^−/−^ mice. MSC transplantation in apoE^−/−^Fas^−/−^ mice decreased the percentage of MDSCs, alleviated the typical atherosclerotic lesions, including atherosclerotic lesions in aortae and liver, and reduced serum cholesterol, triglyceride and low-density lipoprotein levels. MSC transplantation also reduced SLE pathologies, including splenomegaly, glomerular lesions, anti-dsDNA antibody in serum, urine protein and serum creatinine. Moreover, MSC transplantation regulated the generation and function of MDSCs through secreting prostaglandin E 2 (PGE2).

**Conclusion:**

Taken together, these results indicated that the increased MDSCs contributed to atherosclerosis in SLE. MSC transplantation ameliorated the atherosclerosis and SLE through reducing MDSCs by secreting PGE2.

**Graphical Abstract:**

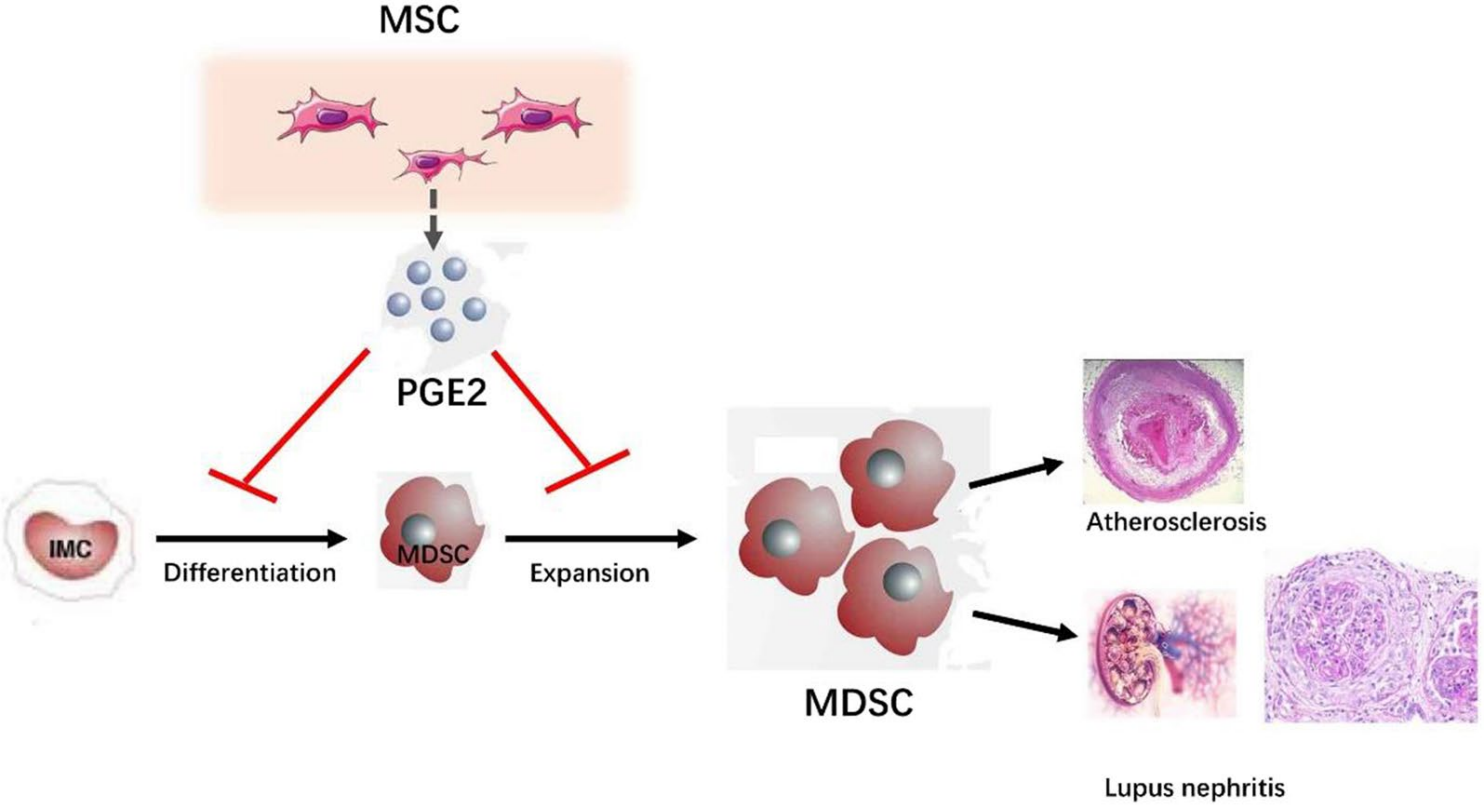

**Supplementary Information:**

The online version contains supplementary material available at 10.1186/s13287-022-03002-y.

## Background

Systemic lupus erythematosus (SLE) is an autoimmune rheumatic disease with a wide spectrum of clinical manifestations, mainly involving kidneys, joints, skin, nervous system and blood [1, 2]. SLE is one of the strongest known risk factors of cardiovascular diseases [3–5]. The atherosclerosis is the main etiology of most cardiovascular diseases [6]. Cardiovascular diseases due to atherosclerosis is currently regarded as one of the leading causes of death among SLE patients [7]. The close relationship between SLE and atherosclerosis is well established. However, up to now, the pathogenesis of atherosclerosis observed in SLE is not fully established.

It is believed that immune dysfunction might contribute to atherosclerosis in SLE. Myeloid-derived suppressor cell (MDSC) is a heterogeneous population of immature hematopoietic cells that have a role in immune tolerance [8, 9]. Accumulating evidences have demonstrated that MDSCs, with suppressive activity, contributed to the negative regulation of immune responses that occurred in many diseases, including cancer [8, 10]. Some studies indicated that MDSCs played pathogenic roles in SLE [11, 12]. Previous study found that the MDSCs were expanded in lupus and exacerbated lupus development through regulating balance of Treg and Th17 cells [13, 14]. It was recently described that the frequencies of MDSCs were elevated in LDLr-/- mice during atherosclerosis [15, 16]. Since MDSC dysfunction is closely related to both SLE and atherosclerosis, respectively, we hypothesized MDSC contributes to the development of atherosclerosis in SLE. In this study, we generated SLE mice prone to atherosclerosis (ApoE^−/−^ Fas^−/−^) and provided direct evidence demonstrating a pathogenic role MDSCs in vivo.

Despite a substantial improvement in diagnosis of atherosclerosis in the setting of SLE over the years, the treatment of atherosclerosis in SLE is still limited. Mesenchymal stem cells (MSCs) are prototypical adult stem cells with the capacity to self-renew and differentiate. MSCs exhibit potent immunomodulatory and immunosuppressive properties [17–19]. Our previous studies have been demonstrated the therapeutic effects of MSCs on various types of autoimmune diseases, including SLE [20–23]. Studies showed that intravascular administration of MSCs reduced atherosclerosis in apolipoprotein E-deficient (ApoE^−/−^) mice and in low-density lipoprotein receptor-deficient (Ldlr^−/−^) mice [24, 25]. We thus hypothesized MSC transplantation is effective for treating atherosclerosis in SLE setting by regulating MDSCs. We tested the hypothesis by performing MSC transplantation in ApoE^−/−^ Fas^−/−^ mice. We further analyzed the therapeutic effects of MSCs on atherosclerosis in SLE and determined the regulatory effects of MSC on MDSC.

## Methods

### Mice and treatment

All animal experiments followed the NIH Guide for the Care and Use of Laboratory Animals and were performed in accordance with guidelines of the Affiliated Drum Tower Hospital of Nanjing University. ApoE^−/−^ mice (B6.129P2-Apoe^tm1Unc^/J) and Fas^−/−^ mice (B6.MRL-Fas^lpr^/J) were purchased from Jackson Laboratory. The genetic background-matched wild-type C57BL/6 J (B6) mice were got from the Model Animal Research Center of Nanjing University. The mice were housed in a pathogen-free environment with a 12 h day/night cycle. To generate mouse model with combination of atherosclerosis and SLE, the ApoE^−/−^ mice were cross-bred with Fas^−/−^ mice, and the resulting heterozygous mice were backcrossed to ApoE^−/−^ mice. Then the resulting ApoE^−/−^ Fas^±^ mice were intercrossed to produce ApoE^−/−^ Fas^−/−^ (AF) mice. Genotyping of wild-type and mutant alleles of apoE and lpr were confirmed by PCR (Additional file 1: Fig. S1). Only female mice were used in the present study. At 20 weeks of age, ApoE^−/−^ Fas^−/−^ mice were fed a high-fat-containing diet (D12109C, Purchased from Changzhou SYSE Bio-Tec.Co.Ltd. China) for 12 weeks. In cases when anesthesia was required, the mice were anaesthetized by intraperitoneal injection with an overdose of pentobarbital sodium (200 mg/kg).

### MSC transplantation

The umbilical cord-derived MSCs were from the Stem Cell Center of Jiangsu Province. The detailed purification and culture procedures of MSCs were described in our previous study [23, 26]. Briefly, after vessels were removed, the umbilical cord was dissected into small sections. The tissues were cultured in Dulbecco’s modified Eagle’s medium with low glucose containing 10% fetal bovine serum. After 10–14 days, the colony of MSC was collected. The MSCs at passages 4–5 were used for transplantation. The dose of MSCs was chosen according to previous study of our group [22]. To determine the therapeutic effects of MSCs on atherosclerosis in SLE, the 30-week-old ApoE^−/−^Fas^−/−^ mice were injected with 5 × 10^5^ MSCs/mouse (*n* = 9) or the same volume of sterilized phosphate-buffered saline (PBS) (*n* = 9) via tail vein, respectively. The eighteen litters of mice were divided randomly into MSC and PBS treatment group. Four weeks after MSC transplantation, the mice were killed and the blood and tissues were harvested for analysis.

### Purification of MSDCs

Mice were anesthetized and killed. The spleens were collected, dissociated, and ground gently in PBS. The splenocytes were strained through a 50-µm mesh to remove clumps. Then, the erythrocytes were lysed with lysis buffer. MSDCs were isolated using mouse MDSC isolation kit (Miltenyi Biotec GmBH, Germany) according to the manufacturer’s protocol.

### Adoptive transfer and elimination of MDSCs

For adoptive transfer of MDSCs, the purified MDSCs were transferred intravenously 10^6^/mouse to the 30 week-old ApoE^−/−^Fas^−/−^ mice via tail vein (*n* = 5). The same volume of sterilized phosphate-buffered saline (PBS) was injected via tail vein as control group (*n* = 5). The 10 litters of mice were divided randomly into MDSCs and PBS treatment group.

For elimination of MDSCs, the 30-week-old ApoE^−/−^Fas^−/−^ mice were injected intraperitoneally (i.p.) with anti-Ly-6G (Gr-1) monoclonal antibody (RB6-8C5) according to a previously reported method [13] (*n* = 5), 250 µg/mouse every 3*d* for total 5 times. The control mice were i.p. with rat IgG2b kappa isotype control antibody (RTK4530) (*n* = 5). The 10 litters of mice were divided randomly into anti Gr-1 and IgG2b treatment group.

### Flow cytometry for MDSCs

In the present study, MDSCs were defined as CD11b + Gr-1 + cells [27]. Cells were isolated from spleen, cervical lymph nodes and blood and resuspended in PBS. The phonotypic profiles of MDSCs were analyzed by staining 1 × 10^5^ cells with allophycocyanin (APC)-conjugated anti-mouse CD 11b (clone M1/70) and phycoerythrin (PE)-conjugated anti-mouse Gr-1 (clone RB6-8C5). Then the fluorescence was measured using a FACS Calibur flow cytometer (BD Biosciences, Mountain View, CA, USA). The FACS data were analyzed by FlowJo software (Treestar, Ashland, USA). The percentage of MDSCs was calculated as a percentage of total splenocytes.

### Serum analysis

Blood was collected from mice with MSC or PBS treatment after overnight fasting. The serum was isolated by centrifugation for analysis.

### Lipid concentrations

Total cholesterol (TC) (A111-1-1), triglyceride (TG) (A110-1-1), low-density lipoproteins (LDLs) (A113-1-1) and high-density lipoproteins (HDLs) (A112-1-1) in serum were determined with enzymatic colorimetric kits from Nanjing Jiancheng Bioengineering Institute (Nanjing, China).

### Alanine aminotransferase, aspartate aminotransferase, creatinine and blood urea nitrogen analysis

The serum alanine aminotransferase (ALT) (C009-2-1), aspartate aminotransferase (AST) (C010-2-1), creatinine and blood urea nitrogen (BUN) (C013-2-1) were measured using kits from Nanjing Jiancheng Bioengineering Institute (Nanjing, China), respectively, as per the manufactures’ instructions.

### ELISA assays for IgG, IgM and IgA and PGEM

The serum IgG (88-50,400-77), IgM (88-50,470-88), and IgA (88-50,450-88) of mice were determined with commercially available ELISA kits (Invitrogen, ThermoFisher Scientific, Waltham, USA). The PGE2 metabolite (PGEM) levels were measured by ELISA kit (Cayman, 514,531) according to the manufacturer’s instructions.

### Anti-dsDNA antibody

The anti-ds-DNA antibodies in serum of mice were determined according to previous studies [28, 29]. The 96-well microtiter plates were coated with 50 µg/ml calf thymus dsDNA (Sigma-Aldrich, D4522) overnight at 4 °C. After blocking with 1% BSA in PBS at room temperature for 2 h, the 200-fold diluted serum (100 µl) was added, followed by incubation at 37 °C for 1.5 h. Then, the goat anti-mouse antibody conjugated to horseradish peroxidase (31,430) in 1:2000 dilution was added, followed by incubation at 37 °C for 1 h. Next, the TMB substrate solution was added to plates, followed by incubation at room temperature for 20 min. Finally, stop solution (1 M H_2_SO_4_) was added and the absorbance of each well was monitored at 450 and 570 nm in a microplate reader.

### Histological analysis

Kidneys harvested at the time of end point were fixed in 4% paraformaldehyde, paraffin-embedded and sectioned. The sections (5 µm) were stained with hematoxylin and eosin (H&E) and periodic acid–Schiff (PAS) for histological analysis.

Livers from mice with different treatments were excised and fixed in 4% paraformaldehyde. Liver specimens were embedded in paraffin, and the tissue sections were prepared and stained with H&E. The histological changes of liver and kidney were evaluated in a blinded fashion by two pathologists.

### Analysis of the atherosclerotic plaques of aortas

The aortas were prepared and analyzed en face. The adipose tissues around the aortas were removed with forceps. Then, the aortas were cut longitudinally along the vessel wall with dissecting scissors. The aortas were stained with Oil Red O according to previous study [28]. Briefly, the aortas were fixed in 4% paraformaldehyde. The fixed aortas were washed three times in distilled water. Then, aortas were immersed in 60% isopropyl alcohol for 5 min. Subsequently, the aortas were incubated in the Oil Red O solution at 37 °C for 60 min in the dark. After staining, the samples of aortas were immersed into 60% isopropyl alcohol, followed by washing with distilled water. The lipid droplets were stained in orange-red, and the other parts were nearly colorless.

### Immunofluorescence

The snap-frozen kidneys were sectioned. Immunofluorescence was performed to detect the deposit of IgG and C3 in the glomeruli of kidney. Frozen sections of kidney were fixed with acetone and incubated with TRITC-goat anti mouse IgG (Jackson ImmunoResearch, 115-025-003) and AlexaFluoe 488-rat anti-mouse C3 (Santa Cruz, sc-58926 AF488) antibody, respectively. All sections were analyzed microscopically with FSX 100 all-in-one microscope (Olympus, Tokyo, Japan).

### Statistical analysis

Data are presented as mean ± SEM. Data were based on three independent experiments. The Student’s t-tests or one-way ANOVA followed by Bonferroni’s test were performed for the normally distributed data. For the non-normally distributed data, the Mann–Whitney *U* test or Kruskal comparison tests were evaluated. All statistical analyses were performed using GraphPad Prism 7 software (GraphPad Software, La Jolla, CA, USA). A *p* value less than 0.05 was considered significantly different.

## Results

### ***ApoE***^***−/−***^***Fas***^***−/−***^*** mice showed atherosclerosis and increased MDSCs***

**Fig. 1:**
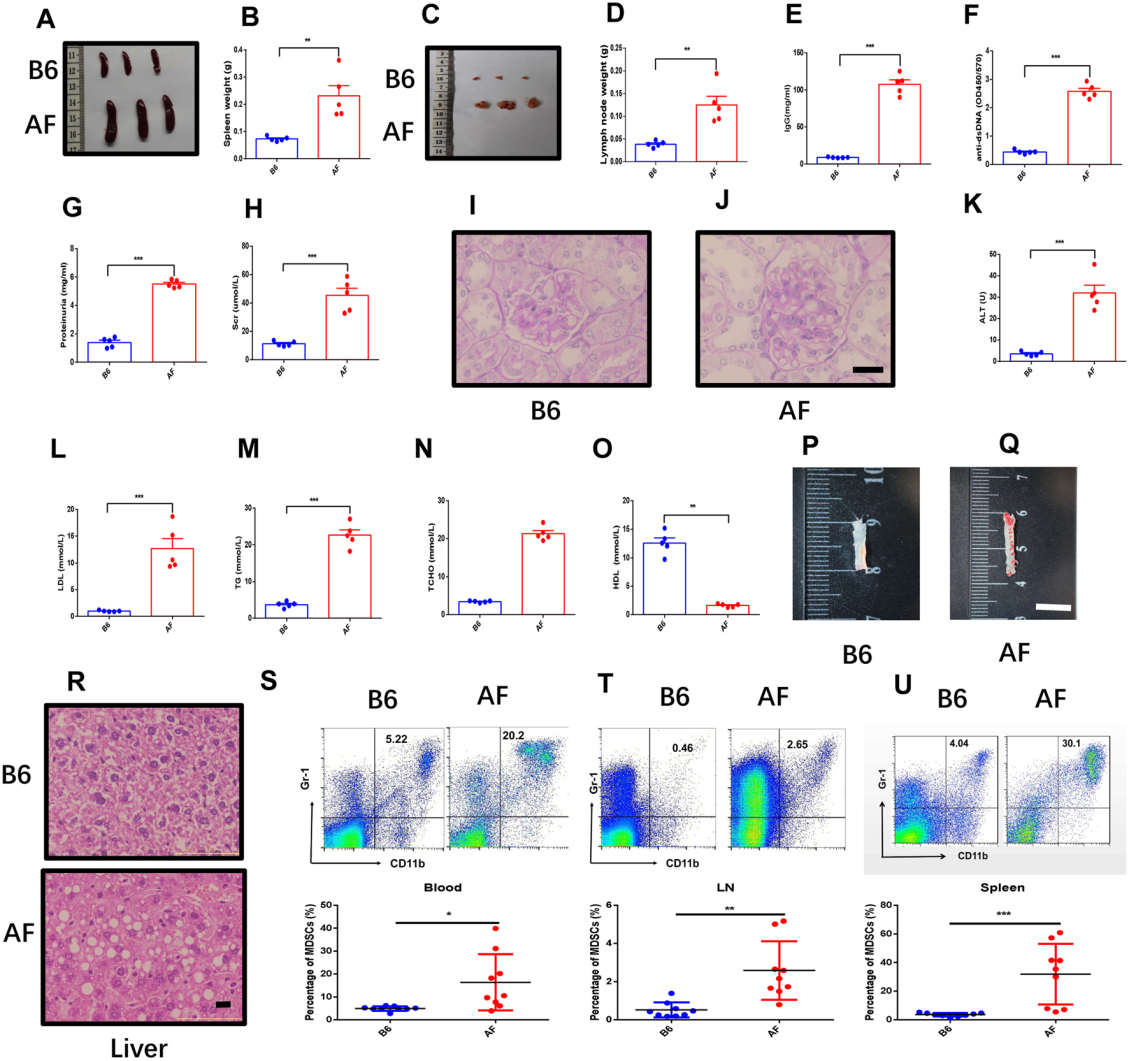
ApoE^−/−^ Fas^−/−^ mice showed atherosclerosis, SLE symptoms and increased MDSCs. Spleens and weights of spleens from B6 and ApoE^−/−^ Fas^−/−^ (AF) mice (**A**, **B**). Cervical lymph nodes and weights of cervical lymph nodes from B6 and ApoE^−/−^ Fas^−/−^ mice (**C**, **D**). Anti-ds DNA antibodies (**E**), proteinuria (**F**), creatinine (**G**) and IgG (**H**) in plasma from B6 and ApoE^−/−^ Fas^−/−^ mice. Representative hematoxylin and eosin (H&E)-stained images of kidney sections from B6 (**I**) and ApoE^−/−^ Fas^−/−^ mice (**J**). The plasma concentration of alanine aminotransferase (ALT) (K), triglycerides (TG) (**L**), total cholesterol (TC) (**M**), low-density lipoprotein (LDL) (**N**), high-density lipoprotein (HDL) (**O**) from B6 and ApoE^−/−^ Fas^−/−^ mice. Aortas from B6 and ApoE^−/−^ Fas^−/−^ mice stained with Oil Red O (**P**, **Q**). Representative H&E -stained images of liver sections from B6 and ApoE^−/−^ Fas^−/−^ mice (**R**). Representative flow cytometry results and percentages of MDSCs of blood (**S**), spleen (**T**) and cervical lymph nodes (**U**) from B6 and ApoE^−/−^ Fas^−/−^ mice. AF, ApoE^−/−^ Fas^−/−^ mice, *n* = 5 mice/group (A–R), *n* = 5 mice/group (S–U). Data were based on three independent experiments. *p < 0.05, **p < 0.01, ***p < 0.001 using Student’s *t*-test

To determine mechanism of atherosclerosis in SLE, we crossed ApoE^−/−^ mice with Fas^−/−^ mice and generated double-mutant ApoE^−/−^Fas^−/−^ mice. The genotypes of mice were identified by PCR (Additional file 1: Fig. S1). The primers for genotyping of ApoE and Fas were showed in Additional file 1: Table S1. We first compared lupus symptoms in ApoE^−/−^Fas^−/−^ mice with B6 mice. There was no difference of body weights between two groups of mice (Additional file 1: Fig. S2A). However, as shown in Fig. 1A, B, there is significant enlargement of spleen, up to 3 times the weight of WT at 30 weeks of age. ApoE^−/−^Fas^−/−^ mice also have enlarged cervical lymph nodes (Fig. 1C, D). The ApoE^−/−^Fas^−/−^ mice displayed the hallmarks of lupus, including high titers of anti-dsDNA antibodies, proteinuria, creatinine, and increased IgG and IgM in serum, which resembled prominent features of human SLE (Fig. 1E–H, Additional file 1: Fig. S2C). However, serum IgA did not significantly increase in ApoE^−/−^Fas^−/−^ mice (Additional file 1: Fig. S2B). Histopathological examination of kidneys from 30-week-old WT and ApoE^−/−^Fas^−/−^ mice demonstrated that the ApoE^−/−^Fas^−/−^ mice displayed a pattern of glomerulonephritis in patients with lupus nephritis, with mesangial cell proliferation, increased glomerular hypercellularity, and thickening of glomerular basement (Fig. 1I–J).

We next detected the typical atherosclerotic lesions in ApoE^−/−^Fas^−/−^ mice. The plasma concentration of ALT (31.92 ± 3.65 U) (Fig. 1K), BUN (34.16 ± 5.82 mmol/L) (Additional file 1: Fig. S2D) and AST (54.70 ± 3.77 U) (Additional file 1: Fig. S2E) in ApoE^−/−^Fas^−/−^ mice was higher than in WT mice (ALT, 3.57 ± 0.45 U, BUN, 6.13 ± 1.15 mmol/L, AST, 6.13 ± 0.23 U). As shown in Fig. 1L–M, the plasma levels of TG (22.61 ± 1.42 mmol/L), TC (21.33 ± 0.82 mmol/L), LDLs (12.7 ± 1.8 mmol/L) significantly increased compared with B6 mice (TG 3.70 ± 0.36 mmol/L, TC 3.44 ± 0.11 mmol/L, LDLs 0.99 ± 0.06 mmol/L), while the HDLs was significantly reduced. Following Oil Red O staining, atherosclerotic lesions were grossly observed in aortic tree of ApoE^−/−^Fas^−/−^ mice (Fig. 1P, Q). Moreover, the accumulation of hepatic lipids in ApoE^−/−^Fas^−/−^ mice, as indicated by H&E and staining was shown in ApoE^−/−^Fas^−/−^ mice (Fig. 1R).

Previous studies have indicated that abnormal of MDSCs was shown in SLE or atherosclerosis, respectively [12, 30–32]; however, the exact role of MDSCs in the atherosclerosis in SLE remains to be elucidated. To determine whether the progression of atherosclerosis in SLE is accompanied with MDSCs, the number of MDSCs were detected by flow cytometry. Compared with WT mice, the frequencies of MDSCs in the blood, spleens and cervical lymph nodes were significantly increased in ApoE^−/−^Fas^−/−^ mice (Fig. 1S). Taken together, these findings suggested that ApoE^−/−^Fas^−/−^ mice showed typical lupus-like symptoms and atherosclerosis accompanied with increasing MDSCs.

### Adoptive transfer of MDSC aggravated atherosclerosis in ApoE^−/−^Fas^−/−^ mice

**Fig. 2:**
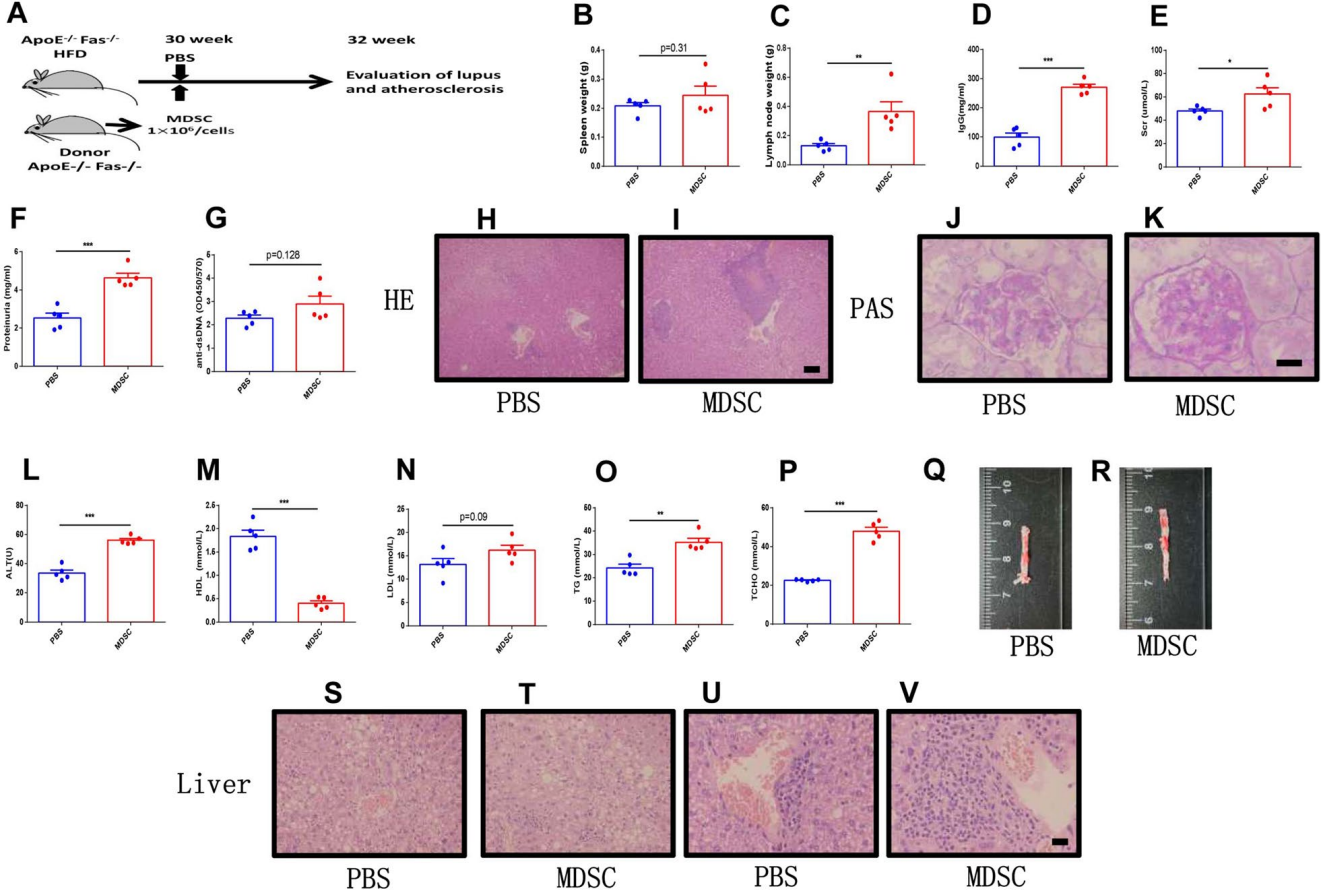
Adoptive transfer of MDSC aggravated atherosclerosis and lupus symptoms in ApoE^−/−^ Fas^−/−^ mice. The ApoE^−/−^Fas^−/−^ mice were transferred with splenic MDSCs or PBS via tail vein. Schematic diagram of adoptively transfer of MDSCs (**A**). The weights of spleens and cervical lymph nodes of ApoE^−/−^ Fas^−/−^ mice (**B**, **C**). IgG (**D**), creatinine (**E**), proteinuria (**F**), and anti-ds DNA antibodies (**G**) in plasma of ApoE^−/−^ Fas^−/−^ mice. Representative H&E and PAS-stained images of kidney sections from ApoE^−/−^ Fas^−/−^ mice (**H**–**K**). The plasma concentration of ALT (**L**), HDL (**M**), LDL (**N**), TG (**O**) and TC (**P**) in ApoE^−/−^ Fas^−/−^ mice. Aortas from ApoE^−/−^ Fas^−/−^ mice stained with Oil Red O (**Q**, **R**). Representative H&E -stained images of liver sections from ApoE^−/−^ Fas^−/−^ mice (**S**–**V**). AF, ApoE^−/−^ Fas^−/−^ mice, *n* = 5 mice/group, Data were based on three independent experiments. *p < 0.05, **p < 0.01, ***p < 0.001 using Student’s *t*-test

To ascertain whether MDSCs play a pathogenic role in the progression of atherosclerosis in SLE, we transferred isolated splenic MDSCs from B6 mice into ApoE^−/−^Fas^−/−^ mice via tail vein (Fig. 2A). The numbers of MDSCs in blood (Additional file 1: Fig. S3A) and spleen (Additional file 1: Fig. S3B) were increased in ApoE^−/−^ Fas^−/−^ mice after transfer of MDSCs. The body weights of ApoE^−/−^ Fas^−/−^ mice showed no significant difference with and without MDSC transfer (Additional file 1: Fig. S4A). The weights of spleens and cervical lymph nodes were higher in MDSC-transferred ApoE^−/−^Fas^−/−^ mice than PBS-treated controls (Fig. 2B, C). Compared with PBS, transfer of MDSCs led to markedly enhanced glomerulonephritis in recipient ApoE^−/−^Fas^−/−^ mice, in which significantly elevated levels of plasma IgG (Fig. 2D), IgA (Additional file 1: Fig. S4B), creatinine (Fig. 2E), proteinuria (Fig. 2G) and BUN (Additional file 1: Fig. S4D). However, the IgM and AST remained unchanged compared to controls (Additional file 1: Fig. S4C and E). The anti-ds DNA antibody increased in MDSC-transferred ApoE^−/−^Fas^−/−^ mice when compared with controls, although this difference was not statistically significant (*p* = 0.128) (Fig. 2F). Lymphocytic infiltrates in the renal vessels were enlarged and glomerular enlargement and damages were exacerbated in ApoE^−/−^Fas^−/−^ mice with MDSC-transfer (Fig. 2H–K).

We next evaluated the characteristics of atherosclerosis in ApoE^−/−^Fas^−/−^ mice with MDSC-treatment. ApoE^−/−^Fas^−/−^ mice with MDSC-transfer had high level of ALT (Fig. 2L). Moreover, the contents of TG, TC and LDLs were pronouncedly exacerbated in ApoE^−/−^Fas^−/−^ mice after adoptive transfer of MDSCs. In contrast, plasma HDLs levels decreased in mice with transfer of MDSCs compared with PBS-treated mice (Fig. 2M–P). Atherosclerotic lesions of aortas, which were shown by lipid-stained areas with Oil Red O staining, were exacerbated in mice with transfer of MDSCs (Fig. 2Q, R). In addition, we found that inflammation infiltration and lipid accumulation in livers were severe in ApoE^−/−^Fas^−/−^ mice with MDSC treatment as determined by H&E staining (Fig. 2S–V). Collectively, our results indicated that adoptive transfer of MDSC aggravated atherosclerosis and lupus symptoms in ApoE^−/−^Fas^−/−^ mice.

### Depletion of MDSCs ameliorated atherosclerosis in ApoE^−/−^Fas^−/−^ mice

**Fig. 3:**
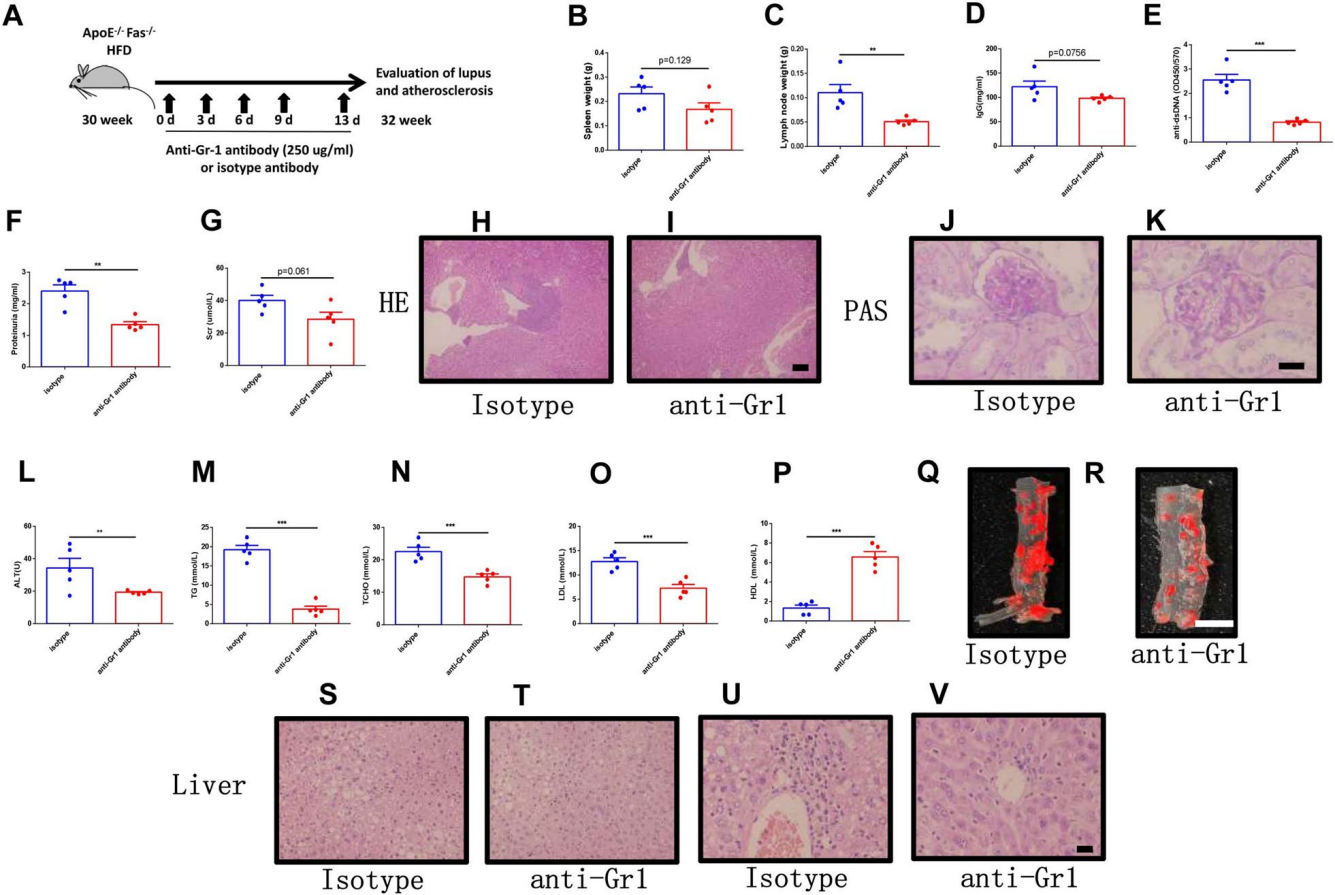
Deletion of MDSCs ameliorated SLE and atherosclerosis in apoE^−/−^Fas^−/−^ mice. The ApoE^−/−^Fas^−/−^ mice were injected with anti-Gr1 or isotype IgG2b antibody, respectively. Schematic diagram of anti-Gr1 antibody treatment (**A**). The weights of spleens and cervical lymph nodes of ApoE^−/−^ Fas^−/−^ mice (**B**, **C**). IgG (**D**), anti-ds DNA antibodies (**E**), proteinuria (**F**), and creatinine (**G**) in plasma of ApoE^−/−^ Fas^−/−^ mice. Representative H&E and PAS-stained images of kidney sections from ApoE^−/−^ Fas^−/−^ mice (**H**–**K**). The plasma concentration of ALT (**L**), TG (**M**), TC (**N**), LDL (**O**) and HDL (**P**) in ApoE^−/−^ Fas^−/−^ mice. Aortas from ApoE^−/−^ Fas^−/−^ mice stained with Oil Red O (**Q**, **R**). Representative H&E -stained images of liver sections from ApoE^−/−^ Fas^−/−^ mice (**S**–**V**). AF, ApoE^−/−^ Fas^−/−^ mice, *n* = 5 mice/group, Data were based on three independent experiments. *p < 0.05, **p < 0.01, ***p < 0.001 using Student’s *t*-test

Since MDSCs aggravated atherosclerosis in SLE in ApoE^−/−^Fas^−/−^ mice, we intend to explore whether deletion of MDSCs alleviate disease in ApoE^−/−^Fas^−/−^ mice. We injected the ApoE^−/−^Fas^−/−^ mice with anti-Gr1 or isotype IgG2b antibody, respectively (Fig. 3A). The numbers of MDSCs in blood and spleen decreased after treatment with anti-Gr1 antibody (Additional file 1: Fig. S5A and B). The results showed that MDSC depletion resulted in reduction of the weights of cervical lymph nodes (Fig. 3B). The weights of spleens also exhibited a tendency to decrease (Fig. 3C). However, the body weights of ApoE^−/−^Fas^−/−^ mice remained unchanged after treatment with anti-Gr1 antibody (Additional file 1: Fig. S6A). We next determined the serum hallmarks of lupus, including anti-ds DNA, IgG, IgA, IgM, proteinuria, Scr and BUN. We found that anti-ds DNA antibody (Fig. 3D) and BUN (Additional file 1: Fig. S6D) were significantly reduced in anti-Gr1 treatment. The proteinuria in anti-Gr1-treated mice (1.34 ± 0.09 mg/ml) was significantly improved compared to isotype IgG2b antibody-treated mice (2.41 ± 0.19 mg/ml) (Fig. 3E). There was also a trend toward decreased IgG and Scr; however, this did not reach significance (Fig. 3F, G). The levels of IgA, IgM and AST remained unchanged after treatment with anti-Gr1 antibody (Additional file 1: Fig. S6B, C and E). Notably, histological analysis showed that anti-Gr1 antibody treatment significantly reduced lymphocytic infiltrates in kidney and glomerulonephritis (Fig. 3H–K). Altogether, these data indicated that lupus-like symptoms were attenuated after MDSC depletion in ApoE^−/−^Fas^−/−^ mice.

We then assessed the atherosclerosis lesion in ApoE^−/−^Fas^−/−^ mice after depletion of MDSC. The serum ALT in anti-Gr1-treated mice (19.30 ± 0.49 U) was significantly improved compared to isotype IgG2b antibody-treated mice (34.38 ± 5.87) (Fig. 3L). Since dyslipidemia play a key role in the progress of atherosclerosis, we next determined the serum TG, TC, LDLs, and HDLs. We observed that TG (3.82 ± 0.77 mmol/L), TC(14.79 ± 0.85 mmol/L), LDLs(6.58 ± 0.55 mmol/L) were significantly decreased in ApoE^−/−^Fas^−/−^ mice after depletion of MDSCs when compared to controls (TG, 19.21 ± 1.12 mmol/L, TC, 22.55 ± 1.35 mmol/L, LDLs, 12.78 ± 0.76 mmol/L) (Fig. 3M–O). Depletion of MDSCs in ApoE^−/−^Fas^−/−^ mice reduced HDLs significantly (6.58 ± 0.55 mmol/L) compared with controls (1.34 ± 0.29 mmol/L) (Fig. 3N). Moreover, the lipid-stained areas in aortas by Oil Red O staining were reduced markedly in ApoE^−/−^Fas^−/−^ mice with anti-Gr 1 antibody treatment (Fig. 3Q, R). Notably, histological analysis by H&E staining showed that depletion of MDSCs in ApoE^−/−^Fas^−/−^ mice significantly reduced lymphocytic infiltrates and lipid accumulation in livers (Fig. 3S–V). All the above observations implied that the depletion of MDSCs in ApoE^−/−^Fas^−/−^ mice ameliorated SLE and atherosclerosis-like symptoms.

### MSC transplantation alleviated lupus symptoms in ApoE^−/−^Fas^−/−^ mice

**Fig. 4:**
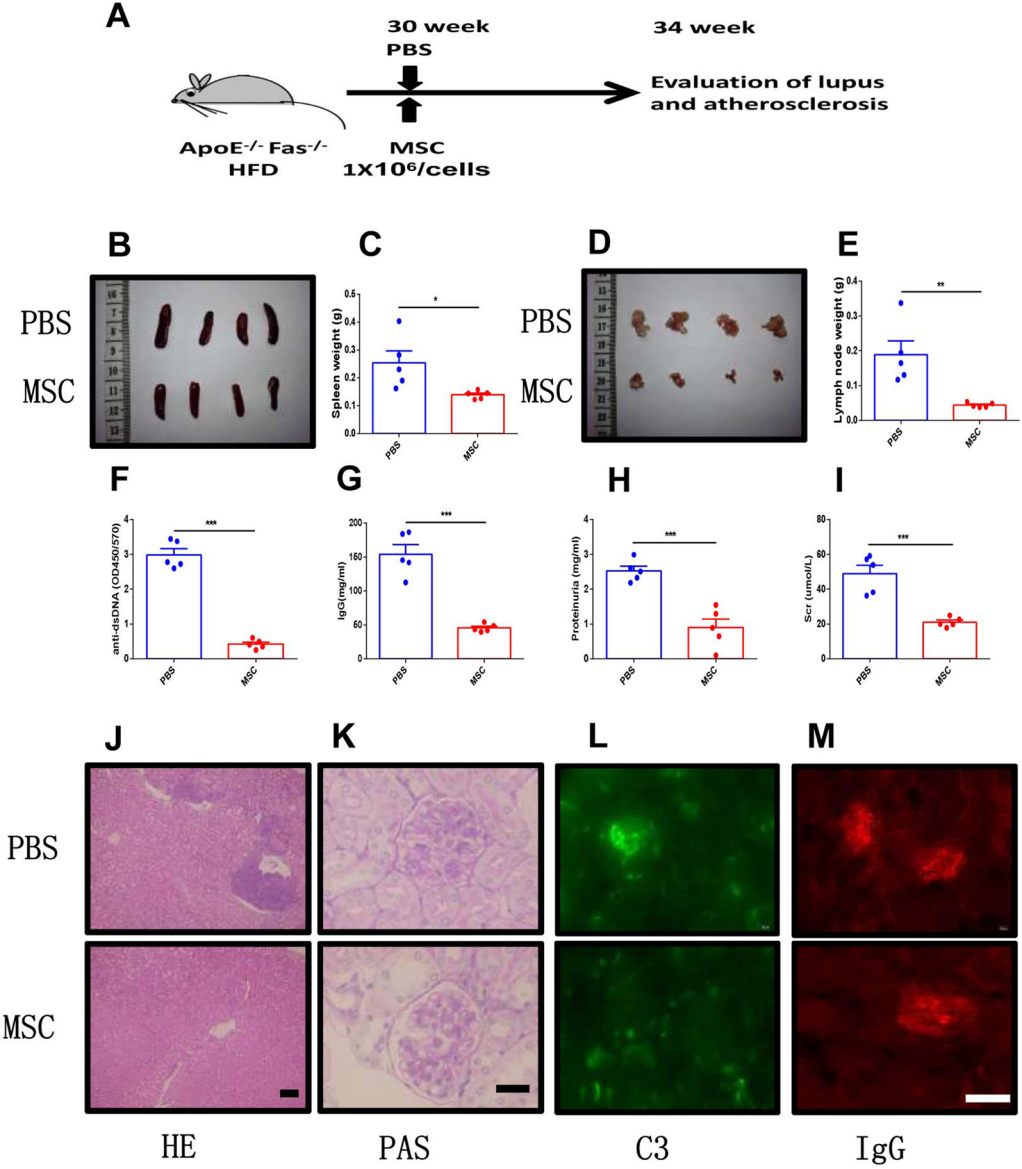
MSC transplantation alleviated lupus symptoms in apoE^−/−^Fas^−/−^ mice. The ApoE^−/−^Fas^−/−^ mice were injected with MSC or PBS via tail vein. Schematic diagram of MSC transplantation (**A**). Spleens and weights of spleens from ApoE^−/−^ Fas^−/−^ mice (**B**, **C**). Cervical lymph nodes and weights of cervical lymph nodes from ApoE^−/−^ Fas^−/−^ mice (**D**, **E**). Anti-ds DNA antibodies (**F**), IgG (**G**), proteinuria (**H**) and creatinine (**I**) in plasma of ApoE^−/−^ Fas^−/−^ mice. Representative H&E and PAS-stained images of kidney sections from ApoE^−/−^ Fas^−/−^ mice (**J**, **K**). Immune staining of deposition of C3 and IgG in kidney sections from ApoE^−/−^ Fas^−/−^ mice (**L**, **M**). AF, ApoE^−/−^ Fas^−/−^ mice, *n* = 5 mice/group, Data were based on three independent experiments. *p < 0.05, **p < 0.01, ***p < 0.001 using Student’s *t*-test

To determine whether MSC transplantation has beneficial therapeutic effects in atherosclerosis in SLE, we injected MSCs into the tail vein in ApoE^−/−^Fas^−/−^ mice (Fig. 4A). We found that MSC transplantation largely reversed splenomegaly and lymphadenectasis in ApoE^−/−^Fas^−/−^ mice (Fig. 4B–E). No significant difference in the body weights were noted between ApoE^−/−^Fas^−/−^ mice with and without MSC transplantation (Additional file 1: Fig. S7A). Since aberrant production of autoantibodies is one of the hallmarks of SLE [28], we next determined the anti-dsDNA antibody and immunoglobulin in the ApoE^−/−^Fas^−/−^ mice. Four weeks after MSC transplantation, the levels of anti-dsDNA antibody in plasma displayed a substantial decrease compared with control group (Fig. 4F). We next examined the plasma IgG, IgM, and IgA in the ApoE^−/−^Fas^−/−^ mice. Compared to control mice, the ApoE^−/−^Fas^−/−^ mice treated with MSC showed a significant decrease of IgG (154.1 ± 13.9 vs 45.6 ± 2.6 mg/ml) (Fig. 4G) and IgA (21.5 ± 1.5 vs 13.4 ± 1.8 mg/ml) (Additional file 1: Fig. S7B). However, no significant difference was found for the plasma IgM between MSC transplantation (21.6 ± 5.2 mg/ml) and control group (29.5 ± 4.2 mg/ml) (Additional file 1: Fig. S7C). Moreover, we observed the downregulation of proteinuria, Scr and BUN levels in ApoE^−/−^Fas^−/−^ mice with MSC transplantation (Fig. 4H, I; Additional file 1: Fig. S7D). Next, we examine the histopathological changes of kidneys from ApoE^−/−^Fas^−/−^ mice with and without MSC treatment. Although ApoE^−/−^Fas^−/−^ mice treated with MSC still showed hyperplasia of glomerular mesangium, the level was lower compared with PBS-treated ApoE^−/−^Fas^−/−^ mice. Additionally, immune cell infiltration into renal interstitial and glomerular enlargement was alleviated in ApoE^−/−^Fas^−/−^ mice with MSC transplantation (Fig. 4J, K). Immune complex deposition in kidneys was shown in SLE patients and model mice [33]. We then examined immunofluorescence for IgG and C3 in mouse kidney cryosections. Fluorescence intensities of IgG and C3 were greatly decreased in ApoE^−/−^Fas^−/−^ mice after MSC transplantation (Fig. 4L, M). Collectively, these data suggested that MSC transplantation have beneficial effects on lupus nephritis in ApoE^−/−^Fas^−/−^ mice.

### MSC transplantation mitigated atherosclerosis in ApoE^−/−^Fas^−/−^ mice

**Fig. 5:**
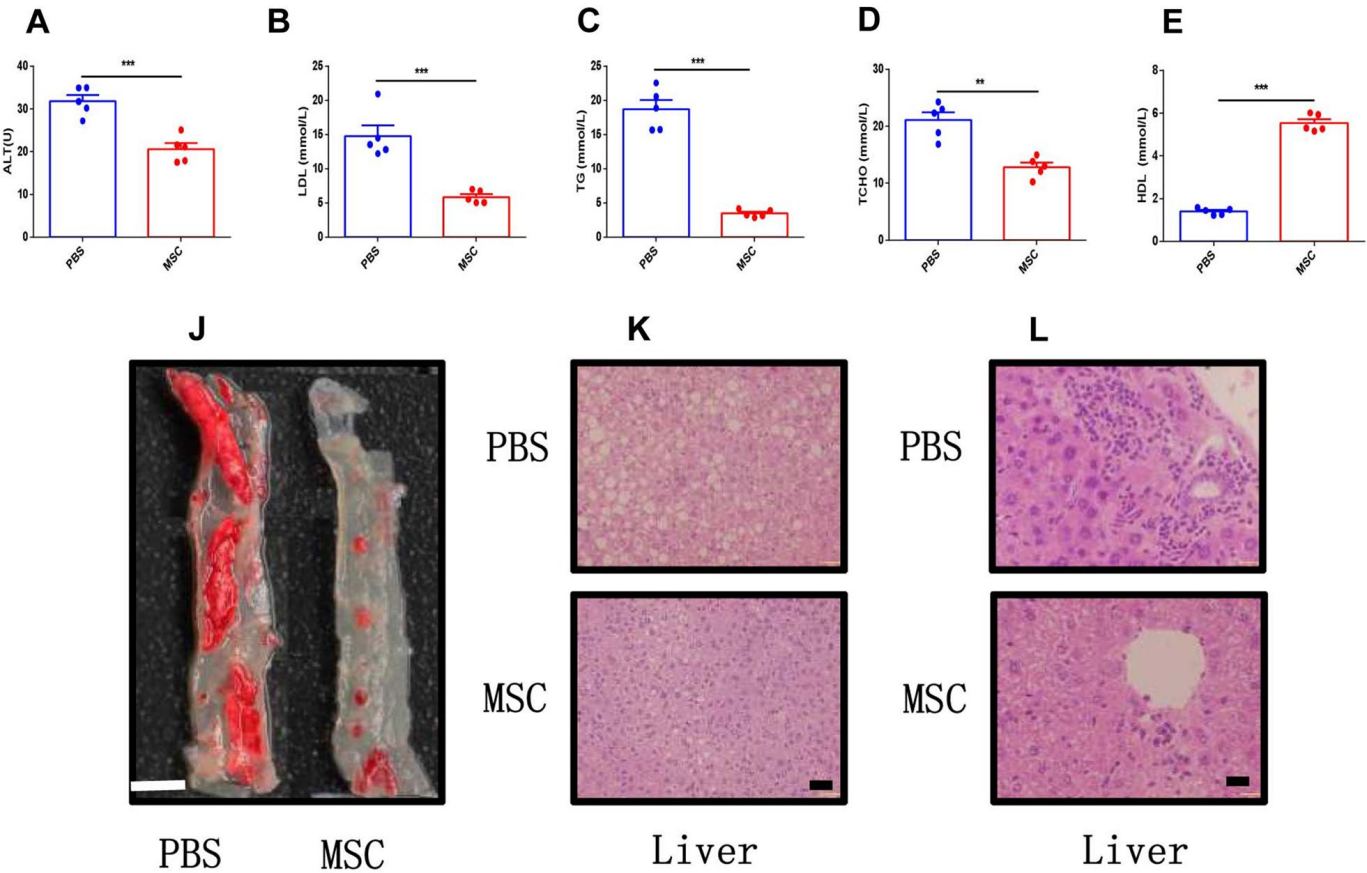
MSC transplantation mitigated atherosclerosis in apoE-/-Fas-/- mice. The ApoE^−/−^Fas^−/−^ mice were injected with MSC or PBS via tail vein. The plasma concentration of ALT (**A**), LDL (**B**), TG (**C**), TC (**D**), and HDL (**E**) in ApoE^−/−^ Fas^−/−^ mice. Aortas from ApoE^−/−^ Fas^−/−^ mice stained with Oil Red O (**J**). Representative H&E -stained images of liver sections from ApoE^−/−^ Fas^−/−^ mice (**K**, **L**). AF, ApoE^−/−^ Fas^−/−^ mice, *n* = 5 mice/group, Data were based on three independent experiments. *p < 0.05, **p < 0.01, ***p < 0.001 using Student’s *t*-test

We next determine the effects of MSC transplantation on atherosclerosis in ApoE^−/−^Fas^−/−^ mice. The serum ALT was significantly lower in ApoE^−/−^Fas^−/−^ mice with MSC transplantation than in those of control mice (20.63 ± 1.38 vs. 31.83 ± 1.48 U) (Fig. 5A). However, these was no significant difference of AST between two groups (Additional file 1: Fig. S7E). Notably, we observed dyslipidemia in ApoE^−/−^Fas^−/−^ mice was pronouncedly improved after MSC treatment, with downregulation of TG (3.44 ± 0.25 vs. 18.71 ± 1.35 mmol/L), TC (12.83 ± 0.80 vs. 21.08 ± 1.38 mmol/L) and LDLs (5.86 ± 0.42 vs. 14.8 ± 1.58 mmol/L) (Fig. 5B–D). However, HDLs was upregulated in ApoE^−/−^Fas^−/−^ mice with MSC transplantation (5.54 ± 0.18 vs. 1.40 ± 0.07 mmol/L) (Fig. 5E). Moreover, we compared the atherosclerotic plaques of aortas in ApoE^−/−^Fas^−/−^ mice with MSC transplantation and control, and found that there is a substantial decrease of lipid-stained areas in aortas in MSC-treated mice (Fig. 5F). More importantly, treatment of mice with MSC significantly reduced lymphocytic infiltrates and lipid accumulation in livers (Fig. 5K, L). Considering all the above results, we concluded that infusion of MSCs attenuated atherosclerosis in ApoE^−/−^Fas^−/−^ mice.

### MSCs reduced MDSCs in ApoE^−/−^Fas^−/−^ mice through PGE2

**Fig. 6:**
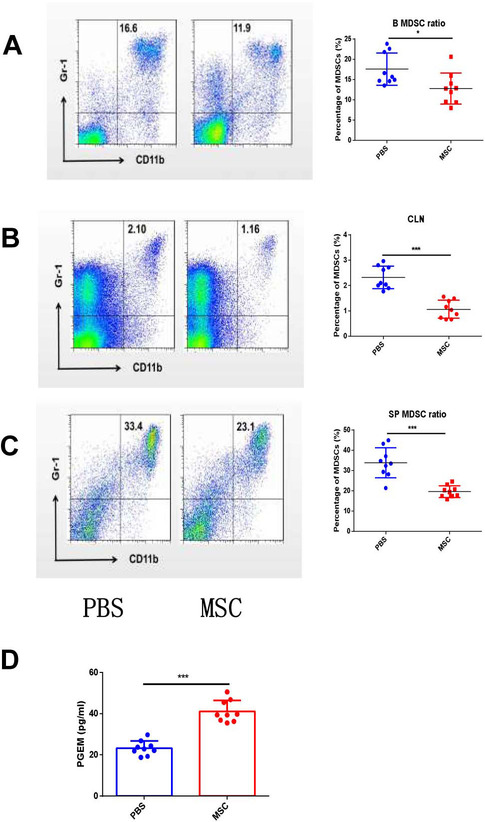
MSCs reduced MDSCs in apoE-/-Fas-/- mice. The ApoE^−/−^Fas^−/−^ mice were injected with MSC or PBS via tail vein. Representative flow cytometry results and percentages of MDSCs of blood (**A**), spleen (**B**) and cervical lymph nodes (**C**) from ApoE^−/−^ Fas^−/−^ mice. The concentration of prostaglandin E metabolite (PGEM) in serum of ApoE^−/−^Fas^−/−^ mice. AF, ApoE^−/−^ Fas^−/−^ mice, *n* = 9 mice/group, Data were based on three independent experiments. *p < 0.05, **p < 0.01, ***p < 0.001 using Student’s *t*-test

To explore the mechanism responsible for the therapeutic effects of MSCs on ApoE^−/−^Fas^−/−^ mice, we next compared the numbers of MDSCs in the mice with and without MSC transplantation. Compared with PBS-treated ApoE^−/−^Fas^−/−^ mice, the frequencies of MDSCs in the blood (12.77 ± 1.28% vs. 17.57 ± 1.33%), spleens (19.60 ± 0.96% vs. 33.80 ± 2.46%) and cervical lymph nodes (1.06 ± 0.12% vs. 2.32 ± 0.15%) were significantly decreased in ApoE^−/−^Fas^−/−^ mice with MSC treatment (Fig. 6A–C). Previous studies have demonstrated that MSC-derived soluble factors contributed to their immunoregulatory activities [34–36]. In our previous study, we have found that PGE2 from culture medium of MSC inhibited the differentiation of MDSCs and polymorphonuclear-MDSCs in vitro. The results also showed that PGE2 from culture medium of MSC increased the expression of Arg-1, gp91^phox^ and iNOS, while decreased the expression of IL-1βin MDSCs in vitro [26]. To verify the regulatory effects of MSC on MDSCs via PGE2 in vivo, we intended to evaluate PGE2 concentration in ApoE^−/−^Fas^−/−^ mice. Since PGE2 is rapidly converted to its metabolite, blood from mice often contains very little PGE2 and measurement of the metabolite is necessary to provide a reliable estimate of PGE2. Therefore, we determined the concentration of PGEM in serum of ApoE^−/−^Fas^−/−^ mice with and without MSC treatment. As excepted, the levels of serum PGE2 were remarkably enhanced in ApoE^−/−^Fas^−/−^ mice with MSC transplantation compared to controls (Fig. 6A–C). These findings indicated that MSCs reduced MDSCs in ApoE^−/−^Fas^−/−^ mice through secreting PGE2.

## Discussion

Accelerated atherosclerosis is a major cause of morbidity and mortality in SLE patients [4, 5]. Risk factors for atherosclerosis in SLE include inflammatory mediators, steroid therapy, and renal injury [3, 6, 7]. There is a need for novel treatment strategies and understanding of mechanisms involved in the pathogenesis of atherosclerosis in SLE. Here, we demonstrated that aberrant MDSCs contributed to atherosclerosis and SLE in model mice of atherosclerosis in SLE (ApoE^−/−^Fas^−/−^ mice). Notably, our findings showed that MSC transplantation ameliorated the atherosclerosis and SLE through reducing MDSCs by secreting PGE2.

Higher risk of cardiovascular disease in SLE patients is mostly related to accelerated atherosclerosis, which leads to clinical symptoms and manifestations compared to the general population [6, 37–39]. Several mouse models which simultaneously exhibited lupus and atherosclerosis have been generated in previous studies [40–42]. In the present study, we generated double-mutant ApoE^−/−^Fas^−/−^ mice by crossing ApoE^−/−^ mice with Fas^−/−^ mice on B6 background. Our results showed that the ApoE^−/−^ Fas^−/−^ mice simultaneously exhibited SLE and atherosclerosis characteristics. In agreement with our results, Feng et al. [40] found that 5 month-old ApoE^−/−^Fas^−/−^ mice displayed lupus-like autoimmunity, including enlarged spleens and lymph nodes, higher levels of IgG and anti-ds DNA antibody, proteinuria, renal damage similar to that found in patients with lupus nephritis. However, in their study, the serum triglycerides, HDL cholesterol, and free fatty acids showed no significant difference between ApoE^−/−^Fas^−/−^ mice and WT mice [40]. The difference between their results and ours is probably as result of the diverse diet. The ApoE^−/−^Fas^−/−^ mice were fed with regular chow diet in study of Feng, however our double knockout B6 mouse was fed with high fat diet. Therefore, the ApoE^−/−^ and Fas^−/−^ double knockout B6 mouse models on high fat diet, as we established here, exhibit both atherosclerosis and lupus-like manifestations and are more suitable for studying the pathogenesis and treatment of accelerated atherosclerosis in SLE.

The reasons responsible for increased risk of atherosclerosis in patients with SLE remains unclear. It is well established that innate as well as adaptive immune response contributed to SLE [43, 44]. Emerging evidences also suggested that components of innate and adaptive immunity were involved in the disease process of atherosclerosis [45, 46]. In recent years, MDSC, which is a universal negative regulator of immune responses, has been recognized as an important contributor in many pathological conditions through regulating innate and adaptive immunity [47, 48]. MDSCs comprise several kinds of immature and mature differentiation stages of myeloid cells. MDSCs possess capacities to suppress immune responses [8–10, 48]. However, the role of MDSCs in progression of SLE and atherosclerosis remains to be elucidated. In our study, we found that expansion of MDSCs of blood, spleen, cervical lymph nodes in ApoE^−/−^Fas^−/−^ mice. This result is consistent with other study, which have shown that SLE patients had a significant increase in MDSCs that correlated positively with disease activity [30]. Study by Ji et al. [13] also showed that PMN-MSDCs were expanded in spleens in the later stages of lupus progression in MRL/lpr mice. Our results also showed that adoptive transfer of MDSCs aggravated atherosclerosis and lupus-like manifestations, whereas elimination MDSCs improves disease progress. Similar observations have also been made, reporting that MDSC depletion markedly attenuated the disease progression in humanized SLE model [30]. These findings may be ascribed to that MDSCs promote the generation of Treg cells, which are critical to the development of immune tolerance, while elimination MDSCs improves the ability of the host’s immune system and improves the efficacy of immunotherapy. However, Park et al. [31] demonstrated that adoptive transfer of MDSCs improved lupus progression by reducing the percentage of effector B cells. As Ji et al. [13] pointed out that the controversial roles of MDSCs in SLE were attributed to the confused phenotypes. Up to now, the percentage and function of MDSCs in atherosclerosis patients and mice are still poorly understood. Our study was consistent with previous study, demonstrating that MDSCs expanded during atherosclerosis [15]. However, study showed that adoptive transfer of MDSCs into LDLr-/- mice fed a western-type diet ameliorated atherosclerosis with 35% by suppression of pro-inflammatory immune responses [15]. In our study, the results indicated that MDSCs played pathogenic roles in ApoE^−/−^Fas^−/−^ mice. The difference between their results and ours is likely due to the finding of Foks’ study [15], indicating that MDSC-mediated suppression could not adequately control immune response during atherosclerosis. However, previous study on SLE found that the suppressive functions of MDSCs in lupus-prone mice was impaired [49]. Taken together, our findings suggested that the adoptive transfer of MDSCs into ApoE^−/−^Fas^−/−^ mice aggravated atherosclerosis and SLE, whereas depleting MDSCs using anti-Gr-1 antibody ameliorated SLE and atherosclerosis in ApoE^−/−^Fas^−/−^ mice.

In addition to stem/progenitor properties, MSCs possess broad immunoregulatory properties and are used to treat immune-based disorders. Our previous studies have demonstrated that MSC infusion is a safe therapy for SLE patients [21, 22]. The development of atherosclerosis was associated with immune responses, including monocyte and macrophage-derived foam cells, infiltrated cells, and inflammatory cytokines [50, 51]. Therefore, MSC was proposed as a novel therapeutic strategy to treat atherosclerosis due to its ability to suppress many components of immune system. However, up to now, no study has reported on MSC transplantation in atherosclerosis in SLE. In the present study, we found that MSC transplantation could restore the number of MDSCs, accompanying with the improvement of symptoms of SLE and atherosclerosis in ApoE^−/−^Fas^−/−^ mice. In agreement with our results, growing evidences demonstrated that MSC transplantation could stabilized the atherosclerotic plaques and had atheroprotective effects [52, 53]. The therapeutic effects of MSC on lupus-like manifestations in ApoE^−/−^Fas^−/−^ mice in this study also confirmed our previous studies which demonstrated that MSCs ameliorated and inhibited disease progression in different lupus models. Collectively, these data suggested that MSC transplantation have beneficial effects on lupus nephritis and atherosclerosis in ApoE^−/−^Fas^−/−^ mice.

In this study, circulating lipoproteins and lipid metabolism in the liver were affected after MSC transplantation. Our results also indicated that depletion of MDSCs by anti-Gr1 antibody could decrease LDL, TG, and TCHO, and increase HDL. The in vitro and in vivo experiment showed that MSCs inhibiting differentiation and expansion of MDSCs. These data collectively serve as a proof that MSCs can be applied for treating atherosclerosis through regulating MDSCs. Several mechanisms have been proposed through which MSCs modulate circulating lipoproteins and ameliorate atherosclerotic lesion. Many lines of evidences have indicated that MSCs regulated lipid metabolism through inhibiting activation of M1 macrophages [54, 55]. In addition, study by Lin et al. [56] has found that MSCs mitigated atherosclerosis through repairing the damaged endothelium and improving endothelial function. Our findings identify that MSCs modulate lipid metabolism through regulating MDSCs. However, further studies are required to understand more about the complex underlying the MSCs transplantation in atherosclerosis.

The beneficial effects of MSC transplantation remain incompletely understood. Generally speaking, the immunosuppressive and anti-inflammatory effects of MSCs were mediated through secreting soluble factors or directly interacting with a variety of immune effector cells [18, 57, 58]. In our study, the results showed success of using MSCs for treating lupus and atherosclerosis, accompanying with decreasing MDSCs. Our previous study showed that culture medium of MSC inhibited MDSC differentiation, which could be abolished by COX2 inhibitor. We also found that PGE2 promoted expression of Arg-1, gp91^phox^ and iNOS and reduced expression of IL-1β in MDSCs. This in vitro study indicated that MSC could inhibit MDSC differentiation and improved its suppressive ability via secreting PGE2 [26]. Consistent with previous in vitro study, we observed that PGE2 was significantly increased after MSC transplantation in ApoE^−/−^Fas^−/−^ mice. Therefore, these data support the conclusion that MSC regulates generation and function of MDSC through secreting PGE2 in ApoE^−/−^Fas^−/−^ mice. It has been well established that PGE2, nitric oxide (NO), transforming growth factor-β (TGF-β), indoleamine 2,3-dioxygenase (IDO) and programmed cell death 1 ligand (PD-L1) are involved in MSC-mediated immunosuppression and therapeutic effects of MSC [4]. Therefore, we think that knockdown or knockout of PGE2 expression in mesenchymal stem cells could partially reverse their therapeutic efficacy in ApoE^−/−^Fas^−/−^ mice. And whether NO, TGF-β, IDO, and PD-L1 were involved in MSC-mediated regulation on MDSC deserves further study.

## Conclusion

In summary, to our knowledge, our data demonstrate for the first time that MDSCs play pathogenic roles in progression of atherosclerosis in SLE, and MDSCs could serve as potential biomarkers and therapeutic targets for treating SLE patients accompanying atherosclerosis. MSC transplantation, which is now used to treat a wide array of diseases, has been confirmed as an effective therapy for mice models with atherosclerosis and SLE manifestations. This study identified a specific mechanism of MSC transplantation, with the ability to inhibit MDSCs through secreting PGE2.

## Supplementary Information

**Additional file 1: Fig. S1.** Genotyping of mice. Mice genotypes of ApoE (**A**) and Fas (**B**) mutation were identified by PCR with DNA obtained by tail biopsy. B6, C57BL/6 mouse, N, negative control, marker, DNA marker, mut, mutation, wt, wild type. **Fig. S2.** The weight, IgA, IgM, BUN and AST in ApoE-/- Fas-/- mice. Weights of B6 and ApoE-/- Fas-/- (AF) mice (**A**). IgA (**B**), IgM (**C**), Blood Urea Nitrogen (BUN) (**D**) and Aspartate Transaminase (AST) (**E**) in plasma from B6 and ApoE-/- Fas-/- mice. AF, ApoE-/- Fas-/- mice, *n*=5 mice/group, **p<0.01, ***p<0.001. **Fig. S3.** Transfer of MDSCs increased the numbers of MDSCs in ApoE-/- Fas-/- mice. The numbers of MDSCs in blood (**A**) and spleen (**B**) after transfer of MDSCs in ApoE-/- Fas-/- mice. **Fig. S4.** The weight, IgA, IgM, BUN and AST in ApoE-/- Fas-/- mice after transfer of MDSCs. The weights of ApoE-/- Fas-/- mice with or without transfer of MDSCs (**A**). IgA (**B**), IgM (**C**), BUN (**D**) and AST (**E**) in plasma from ApoE-/- Fas-/- mice with or without transfer of MDSCs. *n*=5 mice/group, *p<0.05. **Fig. S5.** Anti-Gr1 antibody treatment decreased the numbers of MDSCs in ApoE-/- Fas-/- mice. The numbers of MDSCs in blood (**A**) and spleen (**B**) after treatment with isotype or anti-Gr1 antibody in ApoE-/- Fas-/- mice. **Fig. S6.** The weight, IgA, IgM, BUN and AST in ApoE-/- Fas-/- mice after treatment with anti-Gr1 antibody. The weights of ApoE-/- Fas-/- mice with treatment of isotype or anti-Gr1 antibody (**A**). IgA (**B**), IgM (**C**), BUN (**D**) and AST (**E**) in plasma from ApoE-/- Fas-/- mice with treatment of isotype or anti-Gr1 antibody. *n*=5 mice/group, ***p<0.05. **Fig. S7.** The weight, IgA, IgM, BUN and AST in ApoE-/- Fas-/- mice after MSC transplantation. The weights of ApoE-/- Fas-/- mice with or without MSC transplantation (**A**). IgA (**B**), IgM (**C**), BUN (**D**) and AST (**E**) in plasma from ApoE-/- Fas-/- mice with or without MSC transplantation. n=5 mice/group, *p<0.05, **p<0.01. **Table S1.** Primers for genotyping of ApoE and Fas.

## Data Availability

The data and materials that support the findings of this study are available from the corresponding authors upon reasonable request.

## Abbreviations

MSC: Mesenchymal stem cell
SLE: Systemic lupus erythematosus
MDSCs: Myeloid-derived suppressor cells
PGE2: Prostaglandin E 2
ApoE: Apolipoprotein E
Ldlr: Lipoprotein receptor
B6: C57BL/6 J
WT: Wild type
PBS: Phosphate-buffered saline
i.p.: Intraperitoneally
APC: Allophycocyanin
PE: Phycoerythrin
LDLs: Low-density lipoproteins
HDLs: High-density lipoproteins
ALT: Alanine aminotransferase
AST: Aspartate aminotransferase
Scr: Serum creatinine
BUN: Blood urea nitrogen
PGEM: PGE2 metabolite
H&E: Hematoxylin and eosin
PAS: Periodic acid–Schiff
TG: Triglycerides
TC: Total cholesterol
