# Supplementary material for: Mesenchymal stem cell transplantation alleviated atherosclerosis in systemic lupus erythematosus through reducing MDSCs
Source: Stem Cell Res Ther. 2022 Jul 18;13:328. doi: 10.1186/s13287-022-03002-y (PMC9290280; doi:10.1186/s13287-022-03002-y)

Supplementary Table 1 Primers for genotyping of ApoE and Fas

Gene(mouse)		Primers	Product length (bp)
ApoE mutation	Forward	GCCTAGCCGAGGGAGAGCCG	
	Reverse	TGTGACTTGGGAGCTCTGCAGC	155
ApoE wild type	Forward	GCCTAGCCGAGGGAGAGCCG	
	Reverse	GCCGCCCCGACTGCATCT	245
Fas mutation	Forward	CTCCAGACTCTCTTGCTTTAC	
	Reverse	GACAAGAGATTAGCCTCCAGG	380
Fas wild type	Forward	CTCCAGACTCTCTTGCTTTAC	
	Reverse	GACACCAGTTATGAAGAAAGG	424

Supplementary Figure 1. Genotyping of mice.

Mice genotypes of ApoE (A) and Fas (B) mutation were identified by PCR with DNA obtained by tail biopsy. B6, C57BL/6 mouse, N, negative control, marker, DNA marker, mut, mutation, wt, wild type.

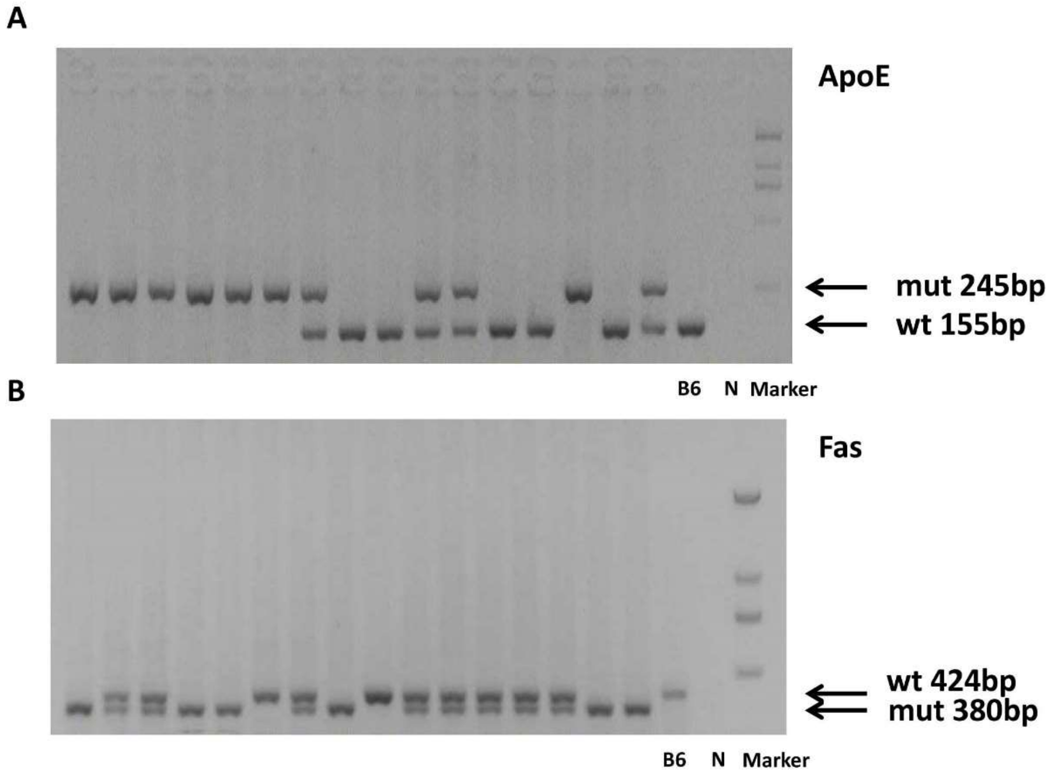

Supplementary Figure 2. The weight, IgA, IgM, BUN and AST in ApoE$^{-/-}$ Fas$^{-/-}$ mice.

Weights of B6 and ApoE$^{-/-}$ Fas$^{-/-}$ (AF) mice (A). IgA (B), IgM (C), Blood Urea Nitrogen (BUN) (D) and Aspartate Transaminase (AST) (E) in plasma from B6 and ApoE$^{-/-}$ Fas$^{-/-}$ mice. AF, ApoE$^{-/-}$ Fas$^{-/-}$ mice, n=5 mice/group, **p<0.01, ***p<0.001

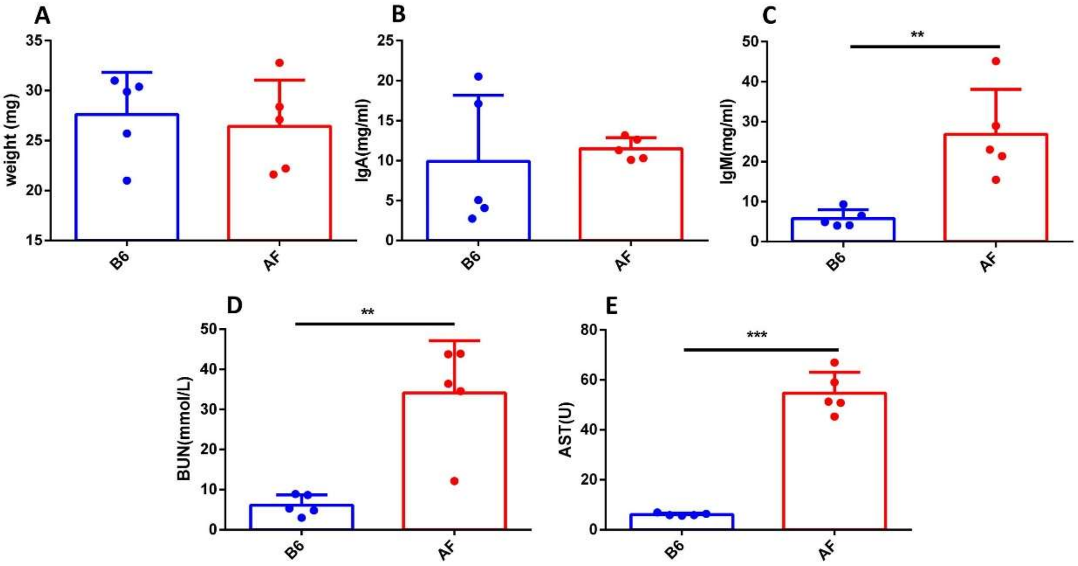

Supplementary Figure 3. Transfer of MDSCs increased the numbers of MDSCs in ApoE$^{-/-}$ Fas$^{-/-}$ mice.

The numbers of MDSCs in blood (A) and spleen (B) after transfer of MDSCs in ApoE$^{-/-}$ Fas$^{-/-}$ mice.

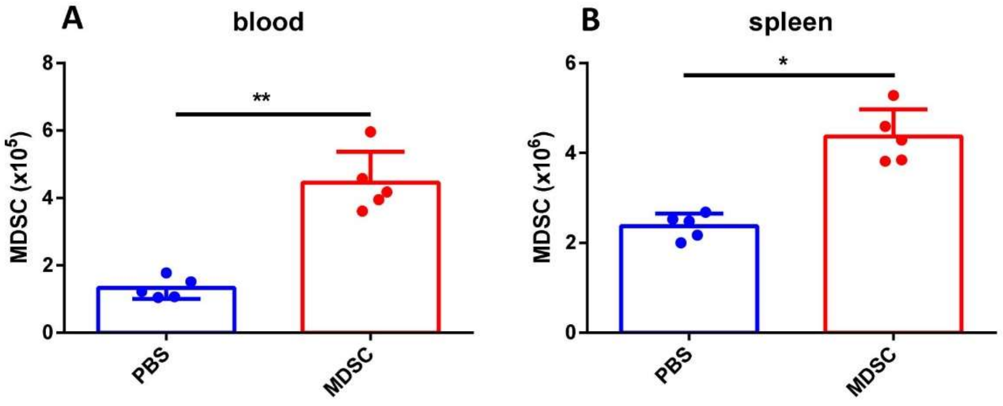

Supplementary Figure 4. The weight, IgA, IgM, BUN and AST in ApoE$^{-/-}$ Fas$^{-/-}$ mice after transfer of MDSCs.

The weights of ApoE$^{-/-}$ Fas$^{-/-}$ mice with or without transfer of MDSCs (A). IgA (B), IgM (C), BUN (D) and AST (E) in plasma from ApoE$^{-/-}$ Fas$^{-/-}$ mice with or without transfer of MDSCs. n=5 mice/group, *p<0.05

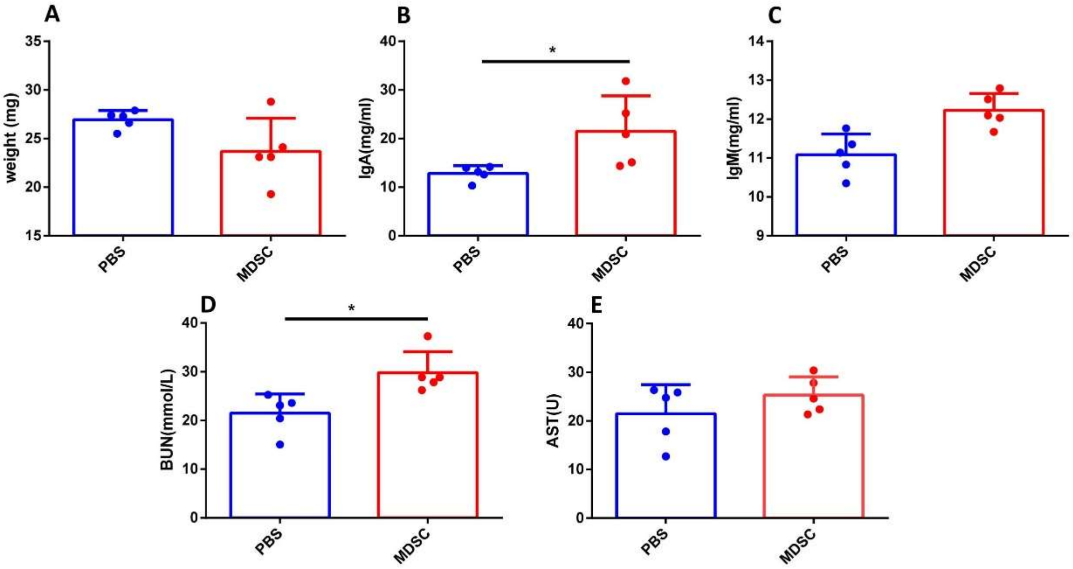

Supplementary Figure 5. Anti-Gr1 antibody treatment decreased the numbers of MDSCs in ApoE$^{-/-}$ Fas$^{-/-}$ mice.

The numbers of MDSCs in blood (A) and spleen (B) after treatment with isotype or anti-Gr1 antibody in ApoE$^{-/-}$ Fas$^{-/-}$ mice.

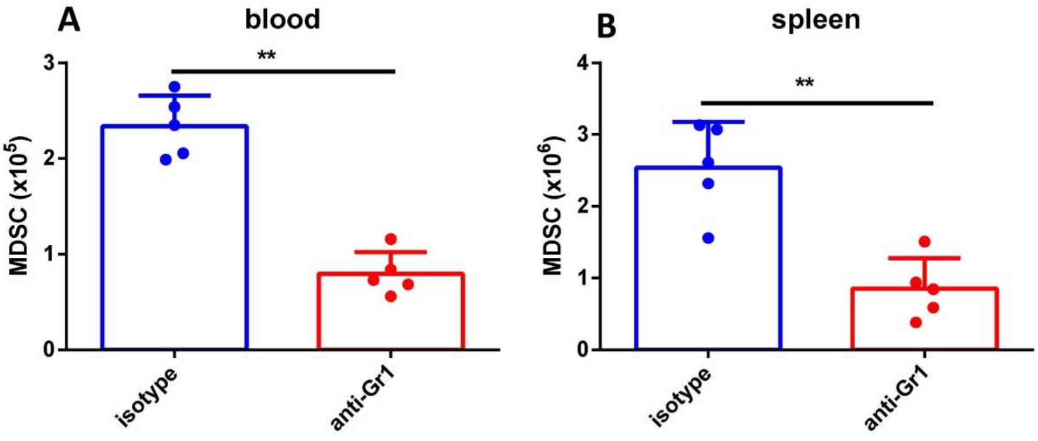

Supplementary Figure 6. The weight, IgA, IgM, BUN and AST in ApoE$^{-/-}$ Fas$^{-/-}$ mice after treatment with anti-Gr1 antibody.

The weights of ApoE$^{-/-}$ Fas$^{-/-}$ mice with treatment of isotype or anti-Gr1 antibody (A). IgA (B), IgM (C), BUN (D) and AST (E) in plasma from ApoE$^{-/-}$ Fas$^{-/-}$ mice with treatment of isotype or anti-Gr1 antibody. n=5 mice/group, ***p<0.05

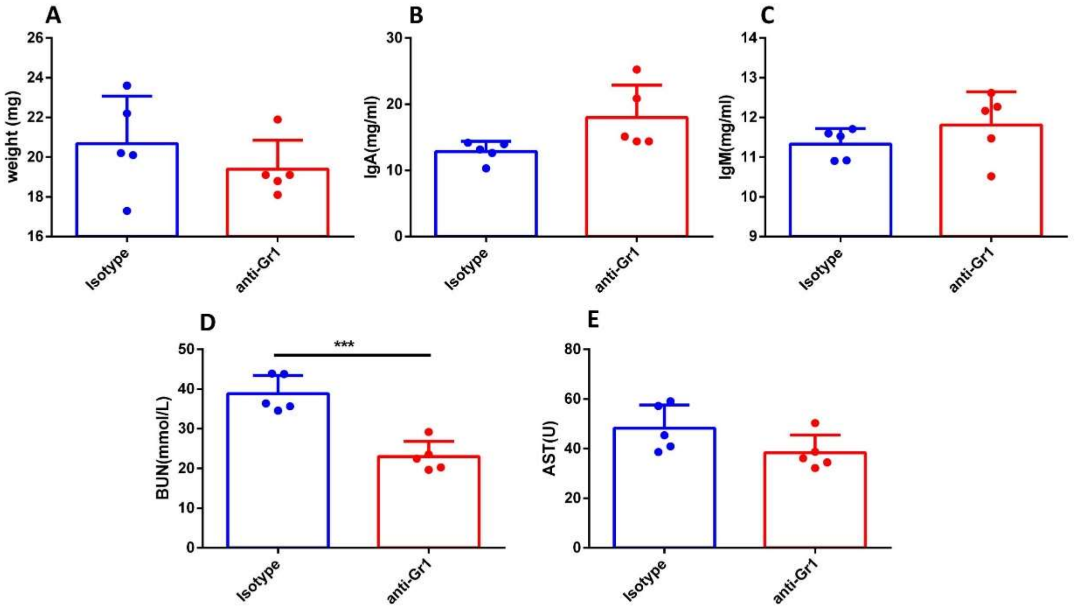

Supplementary Figure 7. The weight, IgA, IgM, BUN and AST in ApoE$^{-/-}$ Fas$^{-/-}$ mice after MSC transplantation.

The weights of ApoE$^{-/-}$ Fas$^{-/-}$ mice with or without MSC transplantation (A). IgA (B), IgM (C), BUN (D) and AST (E) in plasma from ApoE$^{-/-}$ Fas$^{-/-}$ mice with or without MSC transplantation. n=5 mice/group, * p<0.05, **p<0.01

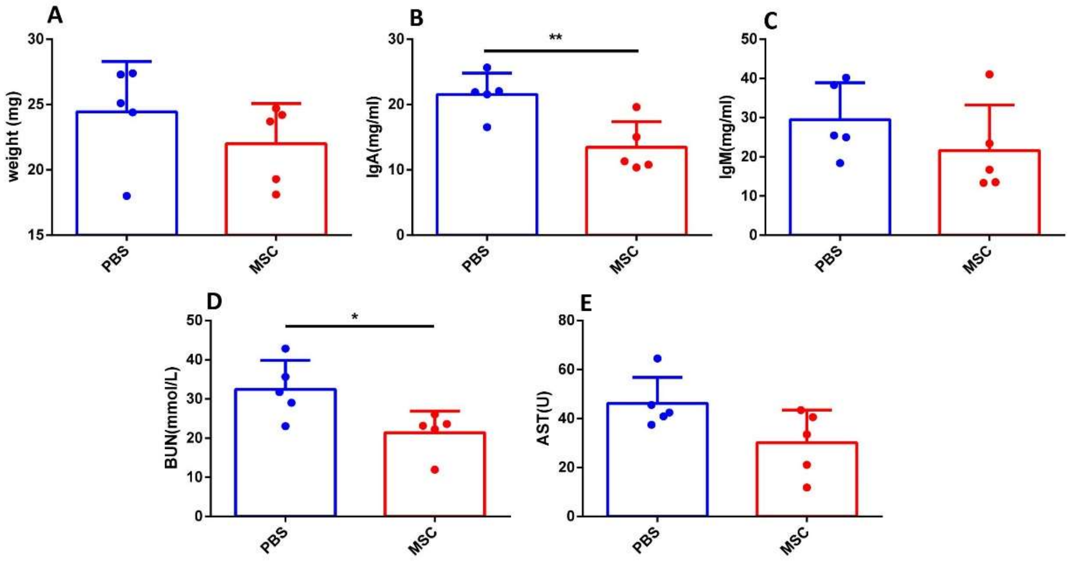

Supplement: Supplementary file 1 — Additional file 1: Fig. S1. Genotyping of mice. Mice genotypes of ApoE (A) and Fas (B) mutation were identified by PCR with DNA obtained by tail biopsy. B6, C57BL/6 mouse, N, negative control, marker, DNA marker, mut, mutation, wt, wild type. Fig. S2. The weight, IgA, IgM, BUN and AST in ApoE-/- Fas-/- mice. Weights of B6 and ApoE-/- Fas-/- (AF) mice (A). IgA (B), IgM (C), Blood Urea Nitrogen (BUN) (D) and Aspartate Transaminase (AST) (E) in plasma from B6 and ApoE-/- Fas-/- mice. AF, ApoE-/- Fas-/- mice, n=5 mice/group, **p<0.01, ***p<0.001. Fig. S3. Transfer of MDSCs increased the numbers of MDSCs in ApoE-/- Fas-/- mice. The numbers of MDSCs in blood (A) and spleen (B) after transfer of MDSCs in ApoE-/- Fas-/- mice. Fig. S4. The weight, IgA, IgM, BUN and AST in ApoE-/- Fas-/- mice after transfer of MDSCs. The weights of ApoE-/- Fas-/- mice with or without transfer of MDSCs (A). IgA (B), IgM (C), BUN (D) and AST (E) in plasma from ApoE-/- Fas-/- mice with or without transfer of MDSCs. n=5 mice/group, *p<0.05. Fig. S5. Anti-Gr1 antibody treatment decreased the numbers of MDSCs in ApoE-/- Fas-/- mice. The numbers of MDSCs in blood (A) and spleen (B) after treatment with isotype or anti-Gr1 antibody in ApoE-/- Fas-/- mice. Fig. S6. The weight, IgA, IgM, BUN and AST in ApoE-/- Fas-/- mice after treatment with anti-Gr1 antibody. The weights of ApoE-/- Fas-/- mice with treatment of isotype or anti-Gr1 antibody (A). IgA (B), IgM (C), BUN (D) and AST (E) in plasma from ApoE-/- Fas-/- mice with treatment of isotype or anti-Gr1 antibody. n=5 mice/group, ***p<0.05. Fig. S7. The weight, IgA, IgM, BUN and AST in ApoE-/- Fas-/- mice after MSC transplantation. The weights of ApoE-/- Fas-/- mice with or without MSC transplantation (A). IgA (B), IgM (C), BUN (D) and AST (E) in plasma from ApoE-/- Fas-/- mice with or without MSC transplantation. n=5 mice/group, *p<0.05, **p<0.01. Table S1. Primers for genotyping of ApoE and Fas. [file 13287_2022_3002_MOESM1_ESM.pdf]
